# The Story of the Insane from Year to Year

**Published:** 1902-05-10

**Authors:** 


### THE STORY OF THE INSANE FROM YEAR TO YEAR.

Fourteenth Annual Series.

### ROYAL ASYLUM, ABERDEEN.

At this asylum the numbers rose from 841 to 887. There were 248 first admissions, 60 readmissions, 136 recoveries, 20 discharged relieved, and 81 died. The average number resident was 867, and the total number of persons under treatment was 1,135. The recoveries stand at 44·1 per cent. of the admissions, and the deaths at 9·3 per cent. on the average number resident, the former being decidedly high and the latter being about the average. Of the year's deaths 21 were caused by cerebral or spinal diseases, 11 by consumption, and 21 by other affections of the lungs. Domestic troubles and mental anxiety are said to account for the insanity in 12 of the admissions, intemperance in drink for 36, hereditary influence for 93, and old age for 21. In 23 cases no cause was known. Large additions to the asylum have been finished and occupied, and the over-crowding entirely removed. These Royal Asylums of Scotland are really combinations of pauper and private asylums. They have for a long time conclusively proved the advantages accruing from this arrangement of treating the classes of patients, and yet it is only within the last few years that any attempt had been made in England to imitate them. At the Aberdeen Asylum private patients pay from £30 to £250 per annum. Paupers are charged £32. About 250 patients are of the private class.

### CITY OF LONDON ASYLUM.

On January 1st the numbers were 460, and on December 31st they had risen to 512. There were 167 first admissions, 17 readmissions, 74 recoveries, 24 discharged relieved, and 31 died. The average number resident during the year was 498, and the total number under treatment was 643. Calculated on the admissions, the recoveries stand at the high percentage of 48·67, and the deaths are only 6·22 per cent. on the average number resident. Of the years' deaths, 15 were caused by cerebral and spinal diseases, 4 by consumption, and the only other thoracic disease proving fatal was heart disease, of which 1 patient died. The insanity of those admitted was attributed to moral causes in 27 cases, to intemperance in drink in 26, to hereditary influence in 39, and in 34 there was no cause known. The greatest number of admissions occurred in July, and the lowest in December. When speaking of the death rate, and remarking on the immunity of the City of London Asylum from colitis, Dr. White says, "its predisposing causes are overcrowding, defective sanitation, and the lowered nerve tone of chronic insanity, and its chief exciting cause is uncontrolled constipation in gross feeders. In some asylums it is endemic, and from time to time becomes epidemic, when it appears to be infections from patient to patient; but seldom affects the staff." If we except Drs. Gemmel and Goodliffe, perhaps no asylum medical officer has more closely studied this disease than Dr. Legge, of Derby, and Dr. Barwise, the Medical Officer of Health, was associated with him in the investigation of one of the epidemics at the Mickleover Asylum. Their report was of an exhaustive nature, and yet a year later, and with more experience of colitis, Dr. Legge had to write—"So far as the cause of the disease is concerned I have to admit that up to the present I have learned little"; and he expressly excludes bad drainage which, he says, was modern at the Derby Asylum, and that there was no reason to suspect it. That bad sanitary arrangements and overcrowding will tend to the spread of this disease is highly probable, if not certain; but that either, or both, has been proved to cause it in our public asylums, we greatly doubt, and we believe Dr. White will one day alter his views on this subject. For many years the attendants and nurses have been trained in this asylum, and a list is given of the names of 60 of the staff who have obtained a certificate, but it does not appear that a three-years' training has preceded the granting of the certificates; still, much good work in that direction has been carried out in the City of London Asylum, and it will continue. Many improvements have been carried out and additions made. Contrasting the old gallery type of asylum with the pavilion plan so much in vogue at present, Dr. White has the following sentence which certainly affords food for reflection: "We have since gone further and further, at ever-increasing cost, towards decentralisation in asylum construction, and the fear now arises whether this has not to a certain extent been at the sacrifice of medical and general supervision, and of facilities and economy of administration." That there are also advantages is hardly to be doubted; but, nevertheless, Dr. White's fears are far from groundless, and when we find that the Home Secretary allows asylums to be built for 2,000 patients, then the fear expressed by him develops into an absolute certainty, and we can only wonder when the system will be altered. The committee record their "appreciation of the services of their medical superintendent." The weekly charge for maintenance is 11s. 1d. for City of London patients, 14s. for out-county cases, and 21s. for private patients.

